# JmjC domain proteins modulate circadian behaviors and sleep in *Drosophila*

**DOI:** 10.1038/s41598-017-18989-1

**Published:** 2018-01-16

**Authors:** Nevine A. Shalaby, Jorge H. Pinzon, Anjana S. Narayanan, Eugene Jennifer Jin, Morgan P. Ritz, Rachel J. Dove, Heike Wolfenberg, Aylin R. Rodan, Michael Buszczak, Adrian Rothenfluh

**Affiliations:** 1 Department of Molecular Biology, University of Texas Southwestern Medical Center, Dallas, TX 75390 USA; 2 Institute for Biology, Freie Universität Berlin, 14195 Berlin, Germany; 3 Department of Psychiatry, University of Texas Southwestern Medical Center, Dallas, TX 75390 USA; 4 Department of Internal Medicine – Division of Nephrology, Department of Human Genetics, University of Utah, Salt Lake City, Utah 84112 USA; 5 Molecular Medicine Program, University of Utah, Salt Lake City, Utah 84112 USA; 6 Department of Psychiatry, Department of Neurobiology and Anatomy, Department of Human Genetics, University of Utah, Salt Lake City, Utah 84112 USA

## Abstract

Jumonji (JmjC) domain proteins are known regulators of gene expression and chromatin organization by way of histone demethylation. Chromatin modification and remodeling provides a means to modulate the activity of large numbers of genes, but the importance of this class of predicted histone-modifying enzymes for different aspects of post-developmental processes remains poorly understood. Here we test the function of all 11 non-lethal members in the regulation of circadian rhythms and sleep. We find loss of every Drosophila JmjC gene affects different aspects of circadian behavior and sleep in a specific manner. Together these findings suggest that the majority of JmjC proteins function as regulators of behavior, rather than controlling essential developmental programs.

## Introduction

All living things, ranging from bacteria to eukaryotes, possess an innate need to respond to environmental conditions that undergo predictable daily oscillations such as light and temperature^1,2^. The circadian clock confers this ability by allowing animals to anticipate these changes and adapt appropriately by adjusting metabolism^3^, hormone secretion^4^ and immunity^5^. In particular, sleep is an essential biological process whose timing is regulated by the circadian clock^6–10^. Circadian regulation of these processes requires parallel, rhythmic fine-tuning of thousands of genes^11–13^ and this coordinated regulation results in an optimal synchronization of physiological processes and behaviors to the time of day. Disruption of circadian rhythms contributes to common metabolic disorders such as obesity and diabetes, and other disorders such as cardiovascular diseases, anxiety, sleep disorders and cancer^14–18^.

Transcriptional control at the level of chromatin is emerging as an important mechanism in the regulation of sleep and circadian rhythms^19–23^. Several studies have shown that histone modifications at specific sites throughout the genome oscillate during the circadian cycle^23–26^, and mutations in chromatin remodeling enzymes such as Brahma or the histone methyltransferase MLL1 alter circadian rhythms^27–29^. The Jumonji (JmjC) protein family includes histone demethylases that act to remove methyl marks off of specific lysine residues on histone proteins, which has a direct impact on chromatin organization and gene expression programs^30,31^. By targeting methyl marks on histone proteins, different JmjC proteins can positively or negatively influence transcription and are expected to serve as key regulators of gene expression in a broad number of contexts. How these family members influence different biological processes remains under investigation.

We hypothesized that fine-tuning of chromatin organization and gene expression programs may critically regulate post-developmental processes such as behavior. To systematically test this model, we took advantage of an established a toolkit, which includes molecularly defined null alleles and tagged transgenic lines for every JmjC family member encoded by the *Drosophila* genome^32^. We tested these *JmjC* mutants in two reproducibly quantifiable behaviors: sleep and circadian rhythms. All 11 mutants tested displayed significant and some distinct changes in circadian rhythms and/or sleep/wake activity. These data indicate that most members of the *Drosophila JmjC* gene family act to fine-tune behavior, many of them in a non-overlapping manner.

## Materials and Methods

### Fly Stocks

All flies were maintained on a standard cornmeal/molasses diet at 25 °C and 75% humidity on a 12-hour day and 12-hour night cycle. The following lines were acquired from the Bloomington Stock Center: *lid*^*10424*^ (BL# 12367) and *lid*^*k06801*^ (BL# 10403). *PSR*^*FM1*^ was provided by Kristin White (Massachusetts General Hospital, Charlestown, MA). The generation and verification of the knockout lines and tagged transgenes is described in Shalaby *et al*.^32^. Flies were outcrossed in groups of >20 for at least 5 generations to the w* Berlin genetic background for behavioral analyses.

### Immunohistochemistry, microscopy and image processing

Adult brains were dissected in 1 × PBS and fixed for 30 min in 4% formaldehyde, washed 3 times, 10 min each, in PBT (1 × PBS, 0.3% Triton-X-100, 0.5% BSA), and incubated in primary antibody diluted in PBT overnight at 4 °C. Next day, samples were washed 3 times, 10 min each, in PBT and incubated in secondary antibodies diluted in PBT at RT for 4 hrs in the dark. Samples were then washed twice in PBT and once in 1 × PBS. TOTO3 was used to stain nuclei. Brains were mounted on a slide with a drop of Vectashield. The following antibodies were used: rat anti-HA 3F10 (Roche, 1:100), Fluorescence-conjugated secondary antibody Alexa488 (Molecular Probes) was used at 1:200. Images were taken using Leica SP8, processed and quantified using Amira 5.3 (Indeed, Berlin, Germany) and Adobe Photoshop CS6.

### Behavioral analysis

**Figure 1:**
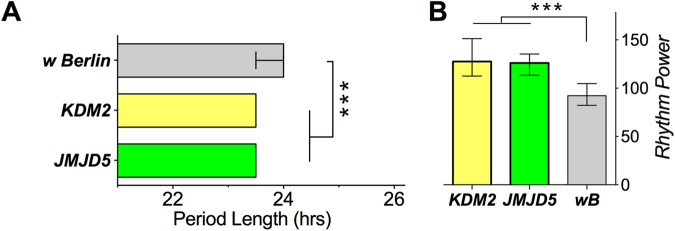
Circadian rhythm phenotypes of *JmjC* genes. (**A**) *KDM2*^*KO*^ and *JMJD5*^*KO*^ flies showed mildly reduced period lengths (estimated effect sizes compared to wild type at −0.74 ± 0.39 95% confidence interval for *KDM2*^*KO*^, and −0.40 ± 0.33 for *JMJD5*^*KO*^ respectively). Note, these effect sizes were estimated assuming normal distributions, which these data were not. Thus, all bar graphs shown in this and following Figures are medians with 95% confidence interval error bars. (Kruskal-Wallis test for multiple comparisons with Dunn’s post hoc adjustment to detect significant differences). (**B**) *KDM2*^*KO*^ and *JMJD5*^*KO*^ flies also showed increased rhythm power compared to *w Berlin* control flies. (*p < 0.05, **p < 0.01, ***p < 0.001; effect sizes = 0.89 ± 0.40 for *KDM2*^*KO*^, 0.77 ± 0.34 for *JMJD5*^*KO*^).

**Figure 2:**
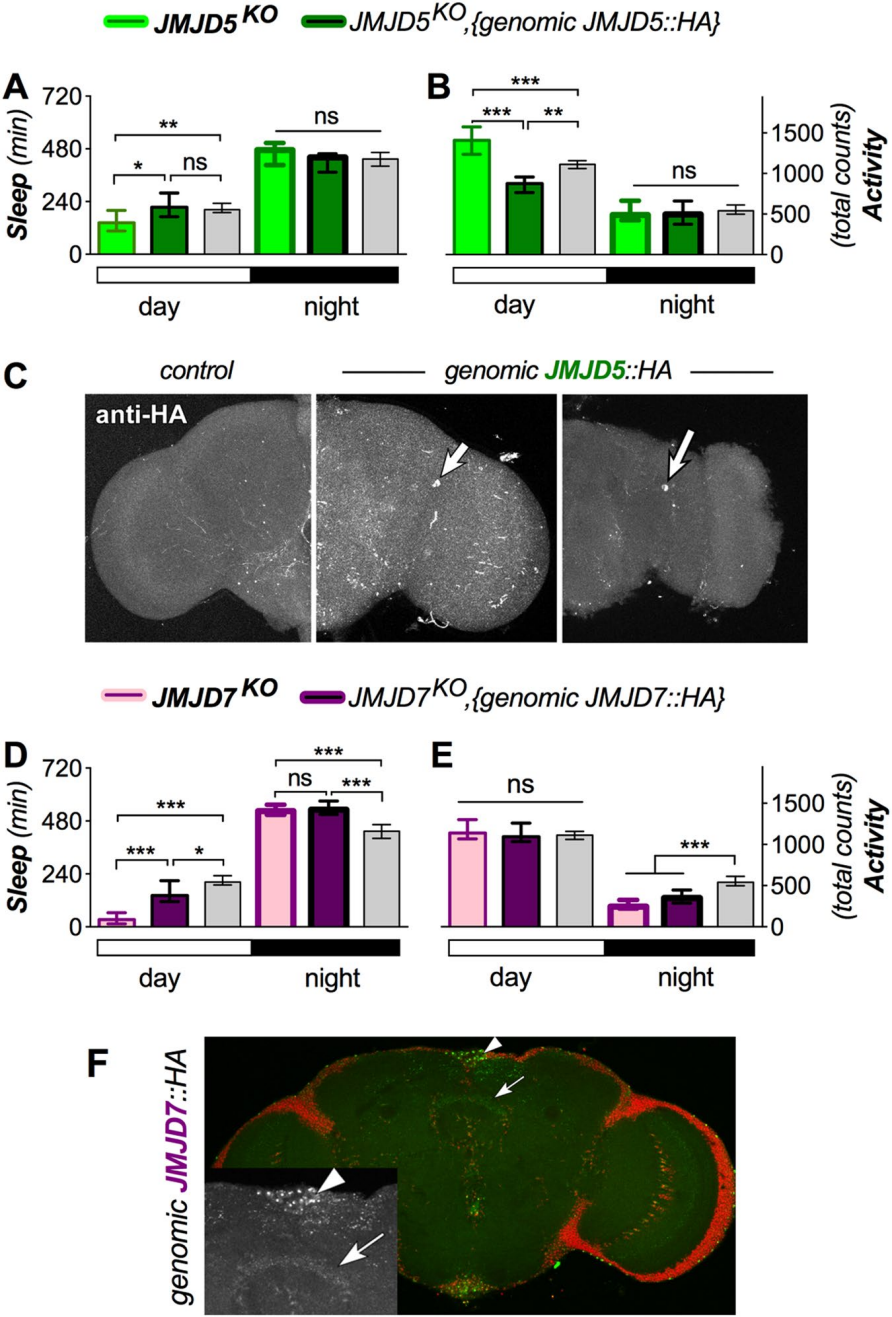
Sleep (and activity) phenotypes of *JMJD5*^*KO*^ and *JMJD7*^*KO*^ flies. (**A**,**B**) *JMJD5*^*KO*^ showed moderately reduced daytime sleep (effect size = −0.68 ± 0.40) concomitant with (**B**) strongly increased daytime activity (effect size = 0.97 ± 40), while nighttime levels of sleep and activity were unaffected (n > 28). In this, and the next two Figures, data from *w Berlin* controls are in grey. The daytime phenotypes are rescued to wild-type levels for sleep, and beyond wild type for activity. (**C**) The *JMJD5::HA* rescue construct (right two brain hemispheres) was expressed in neurons close to the dorsal lateral neurons (arrow). Brains were stained with anti-HA (white) and a negative control is shown at the left. (**D**) *JMJD7*^*KO*^ strongly reduced daytime sleep (effect size = −1.45 ± 0.33 confidence interval), which was partially rescued towards wild-type by expression of the JMJD7::HA transgene (n > 61). The mutant also mildly affected nighttime sleep (effect size = 0.55 ± 0.30). (**E**) The *JMJD7*^*KO*^ mutants also displayed a moderate nighttime activity loss phenotype (effect size = −0.78 ± 0.31). (**F**) The expression pattern (green = anti-HA) of *JMJD7::HA* genomic rescue construct is shown (red = nuclei). Arrowheads point to the pars intercerebralis and arrows point to the fan shaped body, magnified in inset.

**Figure 3:**
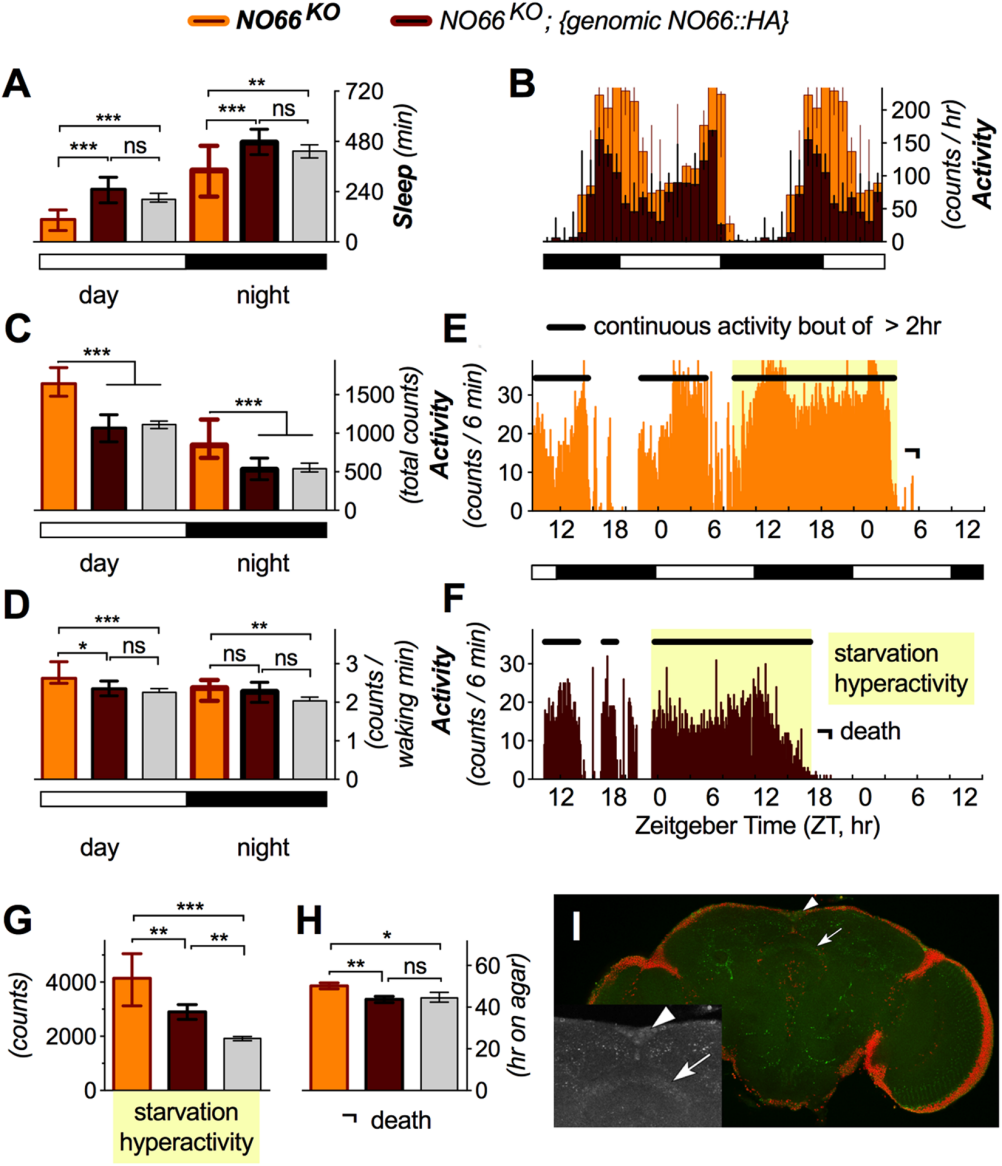
*NO66*^*KO*^ hyperactivity phenotype. (**A**) *NO66*^*KO*^ flies showed a reduction in sleep, strong for the daytime (effect size = 1.10 ± 0.40) and moderate for the night (effect size = 0.66 ± 0.39). This was completely rescued to wild type by expression of the *NO66::HA* genomic transgene (n > 31). (**B**,**C**) Flies also showed a very strong increase in activity, shown for one example fly (B; individual flies shown in this, and the next Figure have values within half a standard deviation from the mean of that genotypic cohort) and the whole cohort. (**C**) The effect size was 1.69 ± 0.43 for daytime activity, and 1.15 ± 0.40 for the night. These phenotypes were also completely rescued by *NO66::HA* expression. (**D**) The hyperactivity phenotype was in part driven by a strong increase in the activity per waking hour (effect size = 1.10 ± 0.40 for daytime waking activity, and 0.66 ± 0.39 at night). The phenotype in daytime waking activity was rescued by *NO66::HA* expression, but rescue flies were no different from mutants or wild type for nighttime waking activity. (**E**–**G**) The hyperactivity phenotype was also evident in the total starvation activity, which was very strongly enhanced (effect size = 1.81 ± 0.35) and partly rescued towards wild type (n > 47). (**E**,**F**) show a single fly example, and (**G**) the whole cohort. (**H**) This increased hyperactivity does not cause premature death; *NO66*^*KO*^ flies even showed a mild delay in starvation-induced death (effect size = 0.41 ± 0.32), which was also rescued by the *NO66::HA* transgene (n > 31). (**I**) The expression pattern (green = anti-HA) of *NO66::HA* genomic rescue construct is shown (red = nuclei). Arrowheads point to the pars intercerebralis and arrows point to the fan shaped body, magnified in inset.

**Figure 4:**
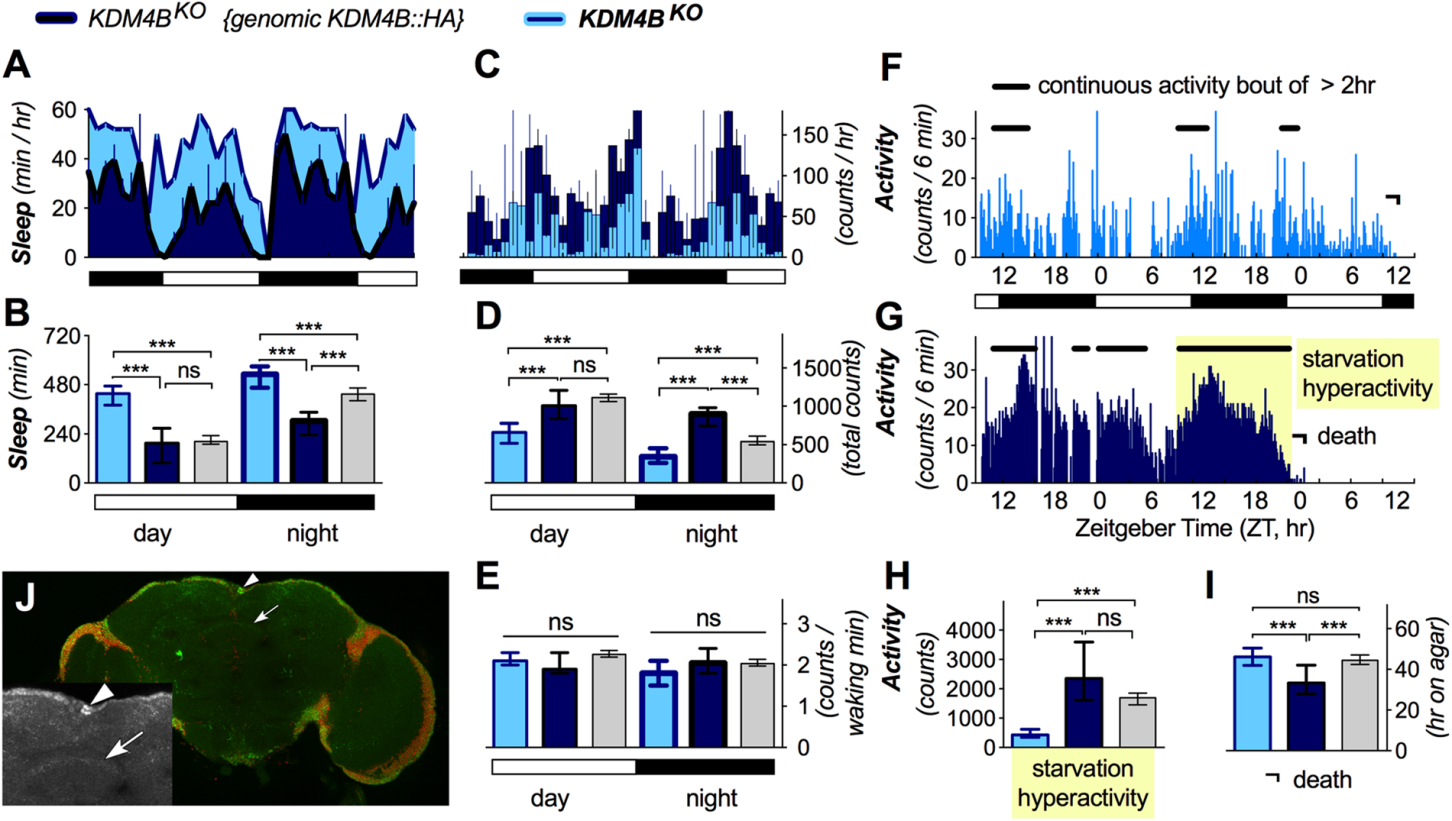
*KDM4B*^*KO*^ sleep phenotype. (**A**–**H**) Shown are mutant (light blue) and rescue (dark) medians with 95% confidence interval. Averages of double-plotted daily sleep of one representative fly (per genotype, **A**) and the two cohorts (**B**) show a strong increase in sleep time in *KDM4B*^*KO*^ (effect size = 1.89 ± 0.46 for daytime sleep, effect size = 0.78 ± 0.42 for night sleep). Average daily activity of one fly each (**C**) and the two cohorts (**D**) indicates a concomitant strong reduction in activity (effect size = −1.79 ± 0.45 for daytime activity, effect size = −0.82 ± 0.42 for night sleep). The daytime sleep and activity phenotypes were rescued to wild type by expressing *KDM4B::HA*, while nighttime sleep and activity were rescued beyond wild-type values. (**E**) The waking activity was not affected in *KDM4B*^*KO*^ mutants compared to wild type (effect sizes = −0.14 ± 0.41 day, −0.41 ± 0.42 night). Individual fly actograms (**F**,**G**) show starvation-induced hyperactivity on non-nutritious agar in rescue flies (**G**), but not in *KDM4B*^*KO*^ mutants (**F**). As a group (**H**), *KDM4B*^*KO*^ flies showed absence of starvation hyperactivity (effect size = −1.22 ± 0.39) but no effect on death (I). Note that in numerous assays shown here, *KDM4B::HA* rescue flies showed opposite phenotypes from *KDM4B*^*KO*^ mutants (as in B,D,H,I; n >31). (**J**) The expression pattern of *KDM4B::HA* genomic rescue construct is shown in (green = anti-HA, red = nuclei) and includes the pars intercerebralis (arrowheads) and fan shaped body (arrows).

**Figure 5:**
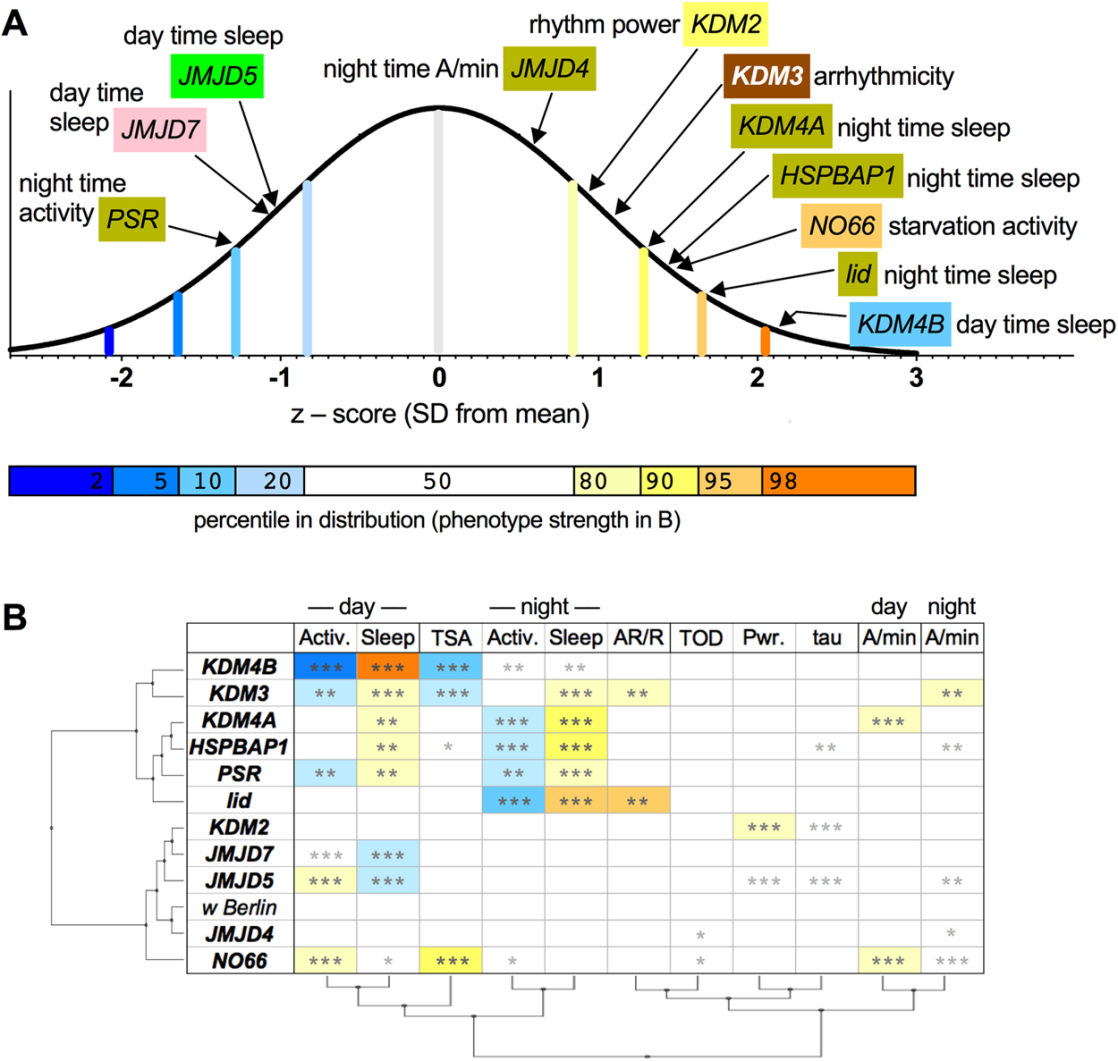
Summary of circadian behavior and sleep phenotypes of *JmjC*^*KO*^ mutants. (**A**) Circadian rhythm, and sleep phenotype strengths of *JmjC*^*KO*^ strains. Phenotype strength (Herge’s *g* effect size) was determined assuming normal distributions and incorporating data from the primary screen and from follow-ups (Figs 2–4). The phenotype strength is shown as the z-score and percentile in an idealized wild-type distribution (color coded, as in Fig. 5B). Note that the means and standard deviations were calculated for each measure in a large w Berlin population (see Supplemental Table 1), but they are idealized, as some measures were not normally distributed (eg. w Berlin total starvation activity). The point is to illustrate the strength of each JmjC mutant’s strongest phenotype, and that few phenotypes are outside the 10th, or 90th percentiles, corresponding to an effect size of >1.3. This is a reflection of i) the lack of “strong” phenotypes, and ii) the considerable variability of the wild-type behavior. (**B**) Phenotype table indicating the behaviors of *JmjC* mutants significantly different from wild type (*p < 0.05, **p < 0.01, ***p < 0.001; Kruskal-Wallis test with Dunn’s post hoc multiple comparison). The strength of those phenotypes is color coded (according to the percentile within the wild-type distribution; see A). Hierarchical clustering dendrograms of these scores are depicted on the left, grouping *JmjC*^*KO*^ mutants, and at the bottom, grouping phenotypic measures. (A/min stands for waking activity per minute; tau for the circadian period length; Pwr. for the power of the rhythm; AR/R for the frequency of arrhythmic flies; TSA for total starvation-induced hyperactivity; and TOD for time of death starting from removal from food).

Sleep and circadian rhythms were assayed as described before^33^. In brief, 2–7 day old flies were entrained for at least 3 days to a 12 hr light: 12 hr dark regimen (LD), and *Drosophila* Activity Monitors (DAM; TriKinetics, Waltham, MA) were used, for circadian rhythm and sleep/activity studies filled with standard food (see above), and for starvation assays filled with 0.7% agar. For sleep/activity studies, we collected data in 2 min bins for 3 days in 12:12 LD cycles, and analyzed them with a custom-written Excel spreadsheet for total sleep time in light and dark, with sleep defined as 6 minutes of uninterrupted inactivity. For circadian rhythms, flies were monitored for 6 days in complete darkness (DD). The data was collected in 30 min bins, and analyzed for rhythm length and strength (peak height above Chi square) using the FaasX software^34^. To determine starvation-induced hyperactivity^35^, which suppresses sleep^36^, flies were placed in DAM on 0.7% agar to provide water. The total cumulative starvation-induced hyperactivity was determined using a custom-written Excel spreadsheet and was defined as the longest continuous activity bout without any interruption by sleep. This normally occurred just prior to the flies’ death (data not shown and^35^). For the primary screen, and for Fig. 1, we assayed each mutant in groups of 16, and repeated the assay at least one time on different days (median n = 31, quartiles: 26–41). Because of its semi-lethality, *lid*^*10424*^*/lid*^*k06801*^ transheterozygous mutants had the lowest n of 16 for some assays. For the follow-up behavioral experiments (Figs 2–4), repeated on separate days, we assayed n = 32–64 mutant and rescue flies. The tables in Fig. 5B and the Supplemental Information was derived from combined primary screen and follow up data.

### Statistics

Data were analyzed in Prism (GraphPad Software, La Jolla, CA). Data from arrhythmicity determinations were dualistic by definition (rhythmic, or not), and were compared by Fisher’s exact test with Bonferroni correction. Half of the period length measures were not normally distributed, while 94/108 other measures passed normality tests (D’Agostino & Pearson normaility test with Bonferroni correction). To achieve highest specificity of our results, we compared all parametric measures by Kruskal-Wallis test with Dunn’s post hoc multiple comparison correction, which does not assume normal distribution. We also analyzed the effect size of the mutants’ phenotypes. Since most distributions were normal, we assumed normal distribution for these effect size estimates, chiefly, in order to determine 95% confidence intervals (for which there is no consensus definition with non-normal data). We determined effect sizes with an Excel spreadsheet (Durham University, UK; http://www.cem.org/effect-size-calculator) that determines Herge’s *g*, a bias-corrected form of Cohen’s *d*, which is essentially the z-score in a normal distribution (Fig. 5; where *d* = 1 means the mutant is one standard deviation away from the wild-type mean), as well as the 95% confidence intervals. Classically, an effect size of >0.5 is considered medium, and >0.8 large. In our experience with behavioral mutants, we consider an effect size of >0.8 a moderate phenotype, >1.3 a strong one, >1.65 a very strong one, and >2.03 an exceptionally strong one; these cutoffs correspond to standing at the following percentiles in a wild-type normal distribution: 20/80, 10/90, 5/95, 2/98. Note that because we can easily assay many individual flies, we have great statistical power (assuming normal distribution). For example, we found 5 genotypes with significantly changed waking activity at night, but only one of those was even of moderate effect size ( > 0.8). With an n of 64, which is easily obtained using just 2 DAM monitors, we have the power to detect effect sizes of 0.5 (alpha = 0.05, beta = 0.8). This explains why we found many significant phenotypes, but considerably fewer strong ones (9/46). Hierarchical clustering was done using the Ward method (www.wessa.net).

### ERG Recordings

ERGs were performed as described in^37^ with the following modifications: Flies were fixed using Elmer’s non-toxic Glue-All. 2 M NaCl was used in the recording and reference electrodes. Electrode voltage was amplified by a Digidata 1440 A, filtered through a Warner IE-210, and recorded using Clampex 10.1 by Axon Instruments. Light stimulus was provided in 1 sec pulses by a computer-controlled white LED system (Schott MC1500). For quantification of depolarization and ‘on’ transients all experiments were carried out in triplicate with at least 10 recordings for each genotype.

## Results

### Several *JmjC* mutants differentially modulate circadian rhythms

A recently established collection of viable *JmjC* knockouts in *Drosophila*, generated using ends-out homologous recombination using an *eyeless*-driven RFP cassette that replaces the open reading frame to create a molecularly-defined null allele^32^, allowed us to test the effect of JmjC-dependent modulation on organism function and behavior in intact animals. Because of the known importance of cyclic transcription in circadian rhythms, we first assayed *JmjC* mutants’ circadian rhythms (the ability of organisms to anticipate and respond to predictable daily oscillations in light and temperature) and sleep/wake activity. First, we examined circadian rhythms in constant darkness to assess endogenous circadian period lengths. Of the 11 viable *JmjC* mutants, seven showed no significant changes, while two exhibited high levels of arrhythmicity (*lid* and *KDM3*^*KO*^), and two others exhibited a subtle shortening of the circadian period length (*KDM2*^*KO*^ and *JMJD5*^*KO*^; Fig. 1A). The latter two mutants also displayed a significant increase in the rhythm power (strength of the rhythm; Fig. 1B).

*KDM3*^*KO*^ mutants showed a high incidence of arrhythmic flies (20 of 78). We were able to rescue the high incidence of arrhythmia (to 7 out of 75 flies; *p* = 0.01, Fisher Exact test) by expressing a tagged genomic *KDM3::HA* transgene generated as part of the *JmjC* toolkit^32^. Anti-HA staining revealed KDM3::HA expression in numerous nuclei (Supplemental Fig. 1), including some close to the dorsal lateral neurons involved in circadian rhythms^38^. Surviving transheterozygotes of previously described *lid* loss-of-function mutants showed a high incidence of arrhythmicity (11/28 of *lid*^*k06801*^*/lid*^*10424*^ transheterozygotes had no discernible rhythms). This phenotype was similar to a previously reported high incidence of arrhythmicity, albeit in *lid*^*10424*^/ + heterozygotes^25^.

### Several *JmjC* mutants differentially modulate sleep and activity levels

We next measured sleep and activity levels in 12hr:12hr light:dark (LD) conditions, to assess sleep duration and locomotion activity. In addition, we assayed mutants’ reaction to starvation on non-nutritious, but hydrating agar. Measures of starvation included both total counts of starvation-induced hyperactivity (and concomitant sleep suppression–as part of a food-searching strategy), as well as time to death^35,36^. Many of the *JmjC* mutants significantly affected sleep and activity levels in LD (see below). We analyzed some of the stronger phenotypes in more detail and rescued them with the genomic tagged *JmjC::HA* rescue transgenes (Figs 2–4). *JMJD5*^*KO*^ showed a reduction in daytime sleep, as well as an increase in daytime activity, both of which could be rescued towards wild-type levels (Fig. 2A,B). JMJD5::HA from the rescue transgene was also expressed in numerous nuclei (Fig. 2C), including some close to the dorsal lateral neurons involved in circadian rhythms^38^.

*JMJD7*^*KO*^ exhibited a reduction in daytime sleep, that could be rescued towards wild type with the genomic-tagged transgene (Fig. 2D,E). The *JMJD7:HA* transgene showed expression in the pars intercerebralis and fan-shaped body (of the adult fly brain), regions known to affect sleep time (Fig. 2 F;^39,40^).

*NO66*^*KO*^ flies exhibited a reduced sleep phenotype and increased activity phenotype (Fig. 3A–D). This was in part driven by an increase in waking activity, i.e. activity per waking minute (Fig. 3D). Upon removal from food, these mutants also showed a strong increase in cumulative starvation-induced hyperactivity (Fig. 3E–G). The enhanced starvation activity did not cause premature death, however. Rather, *NO66*^*KO*^ flies showed a slight delay in starvation-induced death (Fig. 3H). Almost all of these phenotypes were rescued by expressing the genomic *NO66::HA* transgene (Fig. 3A–H), and anti-HA staining showed expression in the pars intercerebralis and parts of the fan-shaped body in the adult fly brain (Fig. 3I).

Lastly, *KDM4B*^*KO*^ exhibited a strong increase in daytime sleep and also significantly more nighttime sleep (Fig. 4A,B), in concert with decreased activity (Fig. 4C,D). Waking activity was not affected, arguing against *KDM4B*^*KO*^ flies being lethargic, or sickly (Fig. 4E). Remarkably, the increased sleep phenotype of *KDM4B*^*KO*^ also extended into their starvation response, and the mutants showed no sign of starvation-induced hyperactivity and arousal from sleep (Fig. 4F–H). A number of these phenotypes could be rescued to wild-type levels by expressing a *KDM4B::HA* transgene, but in some instances, we “rescued” beyond wild type, suggesting possible overexpression effects. The rescuing genomic-tagged transgene was also expressed in the pars intercerebralis and weakly in the fan-shaped body of the adult fly brain (Fig. 4J).

### All *JmjC* non-lethal mutants affect sleep and circadian rhythm

As shown in Fig. 5, all 11 viable *JmjC* mutants tested exhibited significant differences (stars in Fig. 5B) in specific aspects of circadian behaviors, which are described in detail above. Most of the phenotypes observed, however, showed modest z-scores of less than 1.3 (standard deviations away from the mean of wild type; see Fig. 5A and depth of color in Fig. 5B), and effect sizes (Herge’s *g*; Supplemental Table 1). The phenotypes are unlikely to be caused by defective vision, as electroretinogram recordings of all *JmjC* mutants showed normal neurotransmission (Supplemental Fig. 2). However, we identified mutants that specifically affected overall activity (*NO66*^*KO*^), starvation-induced hyperactivity (*KDM3*^*KO*^, *KDM4B*^*KO*^) daytime sleep (*JMJD5*^*KO*^, *JMJD7*^*KO*^ and *KDM4B*^*KO*^), nighttime sleep (*lid*, *PSR*, *KDM4A*^*KO*^ and *HSPBAP1*^*KO*^), rhythm power (*JMJD5*^*KO*^ and *KDM2*^*KO*^), period length (*JMJD5*^*KO*^, *KDM2*^*KO*^*, KDM3*^*KO*^) and overall rhythmicity (*KDM3*^*KO*^, *lid*; Fig. 5B). A hierarchical clustering analysis of this phenotype by genotype space revealed behavioral measures that are affected in concert. This includes, day- (or night-) time sleep and activity: as sleep increases, total activity decreases, we therefore expected these phenotypes to be affected together.

Buoyed by the validity of this clustering along the behavioral axis, we also examined the clustering along the genotype axis, which grouped the *JmjC* genes into two distinct categories, largely correlating with increased, versus decreased amounts of sleep. Mutants of *KDM3*, *KDM4A*, and *KDM4B* all fell into the same category of long-sleepers, and as mentioned above, KDM4A and KDM4B are known to demethylate H3K36me2/3 and all three members are predicted to affect H3K9me2/3 methylation^31^. Furthermore, five out of six genes in this category also affected chromatin^32^. In the second category of short-sleepers, four out of five genes belong to the JmjC domain-only subgroup and all four have recently been shown to act as protein hydroxylases^41,42^.

Taken together, our data indicate that *Drosophila JmjC* genes are involved in the modulation of circadian rhythms and sleep. All non-lethal *JmjC* mutants affect circadian rhythms and/or sleep, from very subtly (*JMJD4*) to very strongly (*KDM4B*). Importantly, none of the measures we assayed were affected by all of the mutants, and no single mutant affected all the measures, implying a certain degree of behavioral specificity for these genes. This is supported by our finding that no mutant tested exhibited the same set of phenotypes as another mutant.

## Discussion

### Most *JmjC* genes regulate circadian rhythms and/or sleep

Using sensitized genetic backgrounds for different signaling pathways, we identified genetic modulation as a mode of action for *JmjC* mutants^32^. To further uncover whether these mutants are required for evolutionarily relevant behaviors, we tested all 11 non-lethal mutants in behavioral assays (sleep, activity and circadian rhythm) that are quantitative and reproducible. To our surprise, we found that all eleven *JmjC* mutants tested significantly altered one or more measures of rhythm and sleep. It had been previously shown that histone modifications at specific sites throughout the genome oscillate during the circadian cycle^23–26^. In addition, a few JmjC proteins have been found to regulate circadian measures/behaviors in various organisms: *KDM2A* knock down in human cells led to a shortening of the period length of cells (as measured by an oscillating luciferase reporter;^43^. We show that *Drosophila KDM2*^*KO*^ also caused a shortening of the period (Fig. 1A). Similarly, loss of *JMJD5/JMJ30*, a JmjC-domain only KDM, caused period-shortening in *Arabidopsis* and in cultured human cells, and the plant and mammalian genes are conserved enough to rescue the phenotype in the reciprocal system^44,45^. We found that *Drosophila JMJD5*^*KO*^ mutation also caused a significantly shortened period-length (Fig. 1A), and in addition reduced daytime sleep (Fig. 2A). Lastly, mammalian Jarid1a, and its *Drosophila* homolog Lid, regulate the expression of the *Period* central clock gene by inhibiting HDAC1 activity, and *Drosophila lid* mutants have been described as highly arrhythmic^25^. We observed similar phenotypes (Fig. 5B), and overall our data indicate that *JmjC* gene function in circadian rhythms is highly conserved from plants to flies to human cells. Additionally, we found that *KDM3*^*KO*^ mutants also have a high incidence of arrhythmicity (Fig. 5B). We therefore newly implicate this histone demethylase, which is involved in gene activation^32,46^, in the regulation of the core clock mechanism.

Much less is known about the involvement of histone modifications and *JmjC* genes in the regulation of sleep. We found that eight of eleven *JmjC* mutants significantly affected the amounts of day- and/or night-time sleep. Half of the phenotypes were less than 1.3 standard deviation (sd) from the mean, yet still highly significant, mainly because of the large number (>30) of individual flies that can be easily assayed. Four mutants showed strong sleep/activity phenotypes (>1.3 sd): *lid*, *KDM4A*^*KO*^, and *HSPBAP1*^*KO*^ all showed increased night time sleep, while *KDM4B*^*KO*^ increased daytime sleep. This latter mutant is particularly intriguing, because not only did it display a very strong phenotype with increased daytime sleep compared to the rescued control (while leaving relative activity per waking minute largely unaffected; Fig. 4A–E), but this increased sleep phenotype also extended into food deprivation. Wild-type flies react to food deprivation with a long bout of starvation-induced hyperactivity, including sleep-suppression, a conserved phenomenon thought to represent foraging for food^35,47^. *KDM4B*^*KO*^ flies lacked starvation-induced arousal, and with it hyperactivity (Fig. 4F–H). This suggests that KDM4B may be generally involved in arousal. Supporting this hypothesis is the finding that our genomic tagged transgene and rescue construct, *KDM4B::HA*, is expressed in the dorsal fan-shaped body (Fig. 4I) strongly resembling a layer receiving dopaminergic input for arousal^40^. It will be interesting to see whether *KDM4B* mutants show disrupted expression of genes involved in dopaminergic transmission, or regulation of Rho-family GTPases, which are also required in the dorsal fan-shaped body for normal sleep^48^.

Contrary to our initial hypothesis, the majority of Drosophila *JmjC* genes are not essential during development^32^. Rather, many of them modulate changes in chromatin organization and gene expression programs^32^. Here we show that many *Drosophila JmjC* genes also modulate behavior, specifically circadian rhythms and sleep. It remains to be determined, whether the phenotypes we observed are a consequence of developmental abnormalities in the nervous system, or whether these genes acutely participate in (cyclic) neuronal function and transcription. We have found that a subset of these JmjC genes is also involved in modulating behavioral responses to ethanol, and that *lid*, *KDM3* and *NO66* are required in the nervous system for normal reactions to ethanol^49^. Together, these data imply that JmjC proteins help to modulate a variety of processes in this organism, including behavior, such as circadian rhythms and sleep.

## Electronic supplementary material

Supplementary Figures
