# Supplementary material for: JmjC domain proteins modulate circadian behaviors and sleep in Drosophila
Source: Sci Rep. 2018 Jan 16;8:815. doi: 10.1038/s41598-017-18989-1 (PMC5770425; doi:10.1038/s41598-017-18989-1)
Supplement: Supplementary file 1 — Supplementary Figures [file 41598_2017_18989_MOESM1_ESM.pdf]

Supplemental Figures for

JmjC domain proteins modulate circadian behaviors and sleep in Drosophila

Nevine A. Shalaby[1,2,*], Jorge H. Pinzon[1,3,*], Anjana S. Narayanan[3,*], Eugene Jennifer Jin[2], Morgan P. Ritz[3], Rachel J. Dove[3], Heike Wolfenberg[2], Aylin R. Rodan[4,5], Michael Buszczak[1,+] and Adrian Rothenfluh[3,5,6,+]

[1]Department of Molecular Biology, University of Texas Southwestern Medical Center, Dallas, TX, 75390, USA

[2]Institute for Biology, Freie Universität Berlin, 14195 Berlin, Germany

[3]Department of Psychiatry, University of Texas Southwestern Medical Center, Dallas, TX, 75390, USA

[4]Department of Internal Medicine – Division of Nephrology, Department of Human Genetics, University of Utah, Salt Lake City, Utah, 84112, USA

[5]Molecular Medicine Program, University of Utah, Salt Lake City, Utah, 84112, USA

[6]Department of Psychiatry, Department of Neurobiology and Anatomy, Department of Human Genetics, University of Utah, Salt Lake City, Utah, 84112, USA

[+] M.B. and A.R. co-supervised the study.

Supplemental Fig. 1. Expression pattern of genomic *KDM3::HA* transgene.

Expression pattern of the *KDM3::HA* rescue transgene, which rescued arrhythmicity in *KDM3*^{KO} flies (see text). Anti-HA staining (blue) is detected in numerous nuclei including in neurons close to the dorsal lateral neurons involved in circadian rhythm. Anti-RFP staining is in red (labeling the *KDM3*^{KO} knock out due to its *3xP3-RFP* expression in the eyes, while not the negative control).

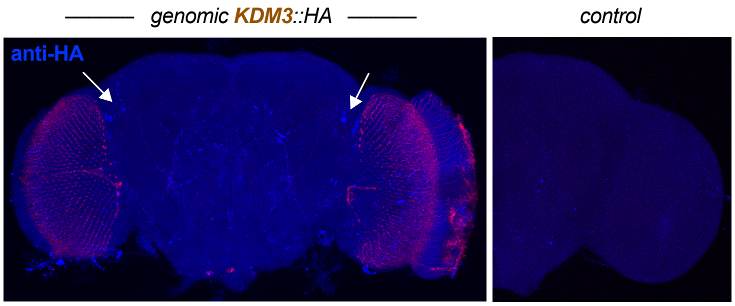
genomic KDM3::HA
control
anti-HA

Supplemental Fig. 2. Electroretinogram (ERG) recordings from *JmjC* mutant flies.

(A) Sample ERG traces from 1 day old flies. On-transient [red circle in control (ctrl) flies] and Depolarization are indicated. Nine out of 11 mutants tested show no significant differences from ctrl flies and exhibit normal photoreceptor responses to light. (B) $KDM3^{KO}$ mutants show a consistent decrease in depolarization, while $HSPBAP1^{KO}$ mutants show increased depolarization, whereas the On-transient remains normal in both cases. (C, D) Bar graphs showing the mean value (± SEM) of On-transients (C) and Depolarization (D) for 15-30 flies per genotype. (* $p<0.0001$).

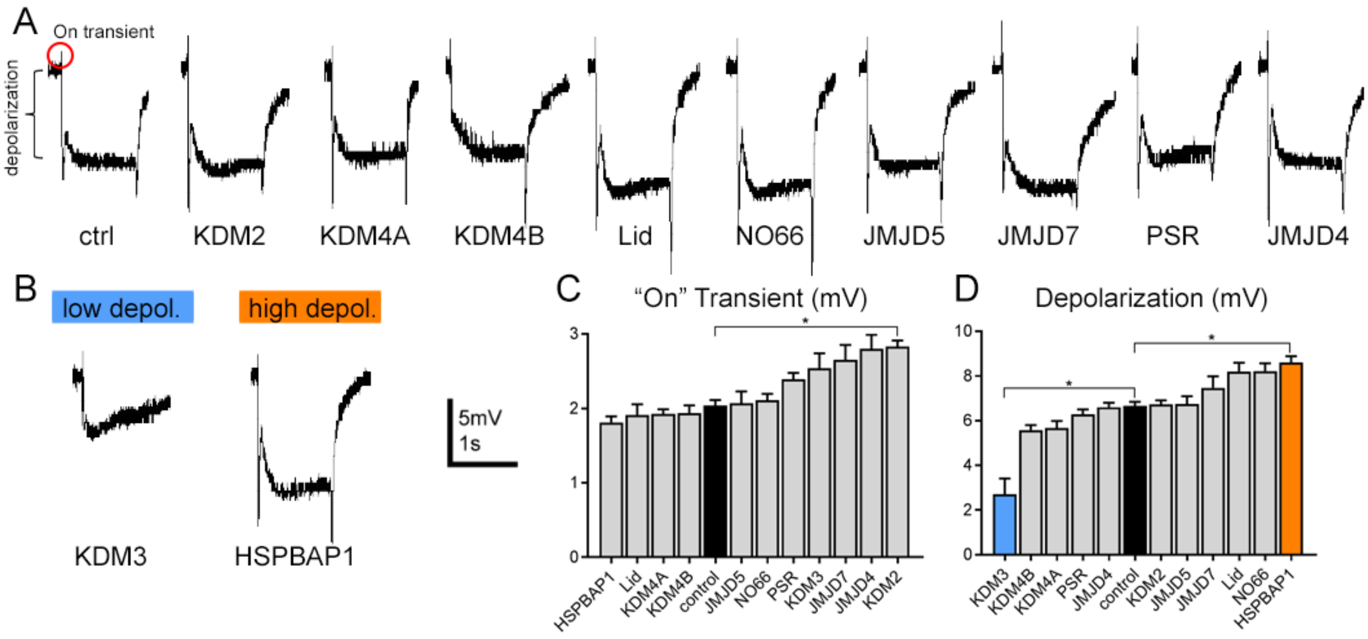
A
On transient
depolarization
ctrl
KDM2
KDM4A
KDM4B
Lid
NO66
JMJD5
JMJD7
PSR
JMJD4
B
low depol.
high depol.
KDM3
HSPBAP1
5mV
1s
C
"On" Transient (mV)
HSPBAP1
Lid
KDM4A
KDM4B
control
JMJD5
NO66
PSR
KDM3
JMJD7
JMJD4
KDM2
D
Depolarization (mV)
KDM3
KDM4B
KDM4A
PSR
JMJD4
control
KDM2
JMJD5
JMJD7
Lid
NO66
HSPBAP1

Supplemental Table 1. Phenotype measures and effect size estimates of *JmjC* mutants.

Shown are the various measures together with the n and standard deviation (sd). The sub-tables are sorted for the effect sizes (Herge's *g*), which are shown with their 95% upper and lower confidence interval (CI). Effect sizes in bold have CIs that do not cross 0 (unadjusted for multiple comparisons).

genotype	Night Sleep (min)	n	sd	Effect Size	Lower CI	Upper CI
lid	625	30	92	**1.60**	1.17	2.03
HSPBAP1	591	60	76	**1.41**	1.08	1.74
KDM4A	586	30	77	**1.30**	0.88	1.72
PSR	580	27	80	**1.24**	0.81	1.68
KDM3	534	43	95	**0.87**	0.51	1.22
KDM4B	505	59	68	**0.68**	0.36	0.99
JMJD7	462	124	147	0.25	0.00	0.49
KDM2	435	25	105	0.05	-0.37	0.48
w Berlin	428	139	128	0.00	-0.24	0.24
JMJD5	399	93	151	-0.21	-0.47	0.06
JMJD4	393	63	141	-0.26	-0.56	0.04
NO66	366	73	159	**-0.45**	-0.73	-0.16

genotype	Night Activity (counts)	n	sd	Effect Size	Lower CI	Upper CI
lid	219	30	179	**-1.38**	-1.80	-0.96
PSR	240	27	189	**-1.29**	-1.73	-0.86
KDM4A	282	30	160	**-1.15**	-1.57	-0.74
HSPBAP1	299	60	247	**-1.06**	-1.39	-0.74
KDM4B	412	59	160	**-0.70**	-1.01	-0.39
KDM2	440	25	251	**-0.54**	-0.97	-0.11
JMJD7	524	132	355	-0.21	-0.45	0.03
w Berlin	590	138	283	0.00	-0.24	0.24
KDM3	676	43	315	0.30	-0.05	0.64
JMJD5	739	93	431	**0.43**	0.16	0.69
JMJD4	760	63	404	**0.52**	0.22	0.82
NO66	855	74	459	**0.75**	0.46	1.04

genotype	Day Sleep (min)	n	sd	Effect Size	Lower CI	Upper CI
KDM4B	443	59	87	**2.10**	1.73	2.47
KDM4A	359	30	141	**1.16**	0.74	1.57
PSR	351	27	122	**1.13**	0.70	1.56
KDM3	347	43	112	**1.11**	0.75	1.47
HSPBAP1	314	60	113	**0.81**	0.50	1.13
lid	268	30	119	0.40	0.00	0.80
w Berlin	223	139	110	0.00	-0.24	0.24
JMJD4	214	63	111	-0.09	-0.38	0.21
NO66	165	73	122	**-0.51**	-0.80	-0.22
KDM2	163	25	94	**-0.55**	-0.98	-0.12
JMJD5	125	93	81	**-0.98**	-1.26	-0.70
JMJD7	98	152	118	**-1.09**	-1.34	-0.85

genotype	Day Activity (counts)	n	sd	Effect Size	Lower CI	Upper CI
KDM4B	654	59	173	**-1.88**	-2.24	-1.52
PSR	818	27	367	**-1.06**	-1.49	-0.64
KDM3	868	43	287	**-0.94**	-1.30	-0.58
lid	944	30	391	**-0.63**	-1.03	-0.23
KDM4A	988	30	393	**-0.48**	-0.88	-0.08
HSPBAP1	1007	60	371	**-0.41**	-0.72	-0.11
w Berlin	1136	138	283	0.00	-0.24	0.24
JMJD4	1197	63	447	0.18	-0.12	0.48
KDM2	1210	25	490	0.23	-0.20	0.66
JMJD7	1443	154	634	**0.61**	0.38	0.85
JMJD5	1475	93	368	**1.06**	0.78	1.34
NO66	1574	74	550	**1.10**	0.80	1.40

Daytime Activity per waking min

genotype	(counts)	n	sd	Effect Size	Lower CI	Upper CI
KDM4A	2.80	30	0.61	**1.09**	0.68	1.51
NO66	2.81	73	0.70	**0.97**	0.67	1.27
KDM3	2.58	26	0.52	**0.67**	0.24	1.09
JMJD5	2.53	61	0.57	**0.52**	0.21	0.82
JMJD4	2.48	63	0.48	**0.44**	0.14	0.74
HSPBAP1	2.47	60	0.56	**0.40**	0.09	0.70
KDM4B	2.45	59	0.55	**0.35**	0.04	0.65
JMJD7	2.47	90	1.19	0.22	-0.05	0.49
w Berlin	2.29	139	0.43	0.00	-0.24	0.24
PSR	2.18	27	0.53	-0.24	-0.65	0.18
lid	2.12	30	0.76	-0.33	-0.72	0.07
KDM2	2.13	25	0.59	-0.34	-0.77	0.09

Nighttime Activity per waking min

genotype	(counts)	n	sd	Effect Size	Lower CI	Upper CI
KDM3	2.62	26	0.78	**1.06**	0.63	1.50
NO66	2.48	73	0.69	**0.77**	0.48	1.06
JMJD5	2.43	61	0.59	**0.72**	0.41	1.03
HSPBAP1	2.45	60	0.74	**0.69**	0.38	1.00
JMJD4	2.45	63	0.97	**0.59**	0.29	0.89
lid	2.26	29	0.84	0.36	-0.04	0.77
KDM4A	2.23	30	0.84	0.31	-0.09	0.70
PSR	2.18	27	0.53	0.25	-0.16	0.66
JMJD7	2.17	91	0.90	0.15	-0.11	0.42
KDM2	2.13	25	0.59	0.14	-0.28	0.57
w Berlin	2.06	139	0.46	0.00	-0.24	0.24
KDM4B	1.92	59	0.47	**-0.30**	-0.61	0.00

Total Starvation Activity						
genotype	(counts)	n	sd	Effect Size	Lower CI	Upper CI
KDM4B	493	63	354	**-1.38**	-1.68	-1.08
KDM3	902	28	556	**-0.92**	-1.32	-0.51
HSPBAP1	1235	31	682	**-0.61**	-1.00	-0.23
KDM4A	1432	32	858	**-0.43**	-0.80	-0.06
JMJD5	1472	32	706	**-0.40**	-0.77	-0.03
JMJD7	1840	30	945	-0.07	-0.45	0.31
JMJD4	1902	31	1019	-0.01	-0.39	0.36
w Berlin	1917	211	1155	0.00	-0.19	0.19
PSR	2114	16	1559	0.17	-0.34	0.67
KDM2	2177	31	1052	0.23	-0.15	0.60
lid	2568	16	1697	**0.54**	0.03	1.05
NO66	3982	79	2002	**1.44**	1.15	1.72

Time of Death on Agar						
genotype	(hours)	n	sd	Effect Size	Lower CI	Upper CI
JMJD4	39.4	31	11.7	**-0.57**	-0.96	-0.19
lid	39.8	16	10.0	**-0.55**	-1.06	-0.03
JMJD7	42.0	30	6.9	-0.35	-0.74	0.03
KDM4A	42.3	32	9.0	-0.32	-0.69	0.05
KDM2	43.2	31	10.2	-0.23	-0.61	0.15
HSPBAP1	43.3	31	11.3	-0.22	-0.60	0.15
KDM3	43.6	28	8.6	-0.20	-0.59	0.20
JMJD5	44.7	32	8.2	-0.10	-0.47	0.27
w Berlin	45.8	211	11.0	0.00	-0.19	0.19
KDM4B	49.7	63	14.1	**0.34**	0.05	0.62
NO66	50.5	79	12.5	**0.42**	0.15	0.68
PSR	50.6	16	12.8	0.44	-0.07	0.95

genotype	Period Length (hr)	n	sd	Effect Size	Lower CI	Upper CI
KDM2	23.47	36	0.78	**-0.78**	-1.18	-0.39
HSPBAP1	23.63	43	0.54	**-0.64**	-1.00	-0.27
JMJD5	23.55	58	1.53	**-0.48**	-0.81	-0.15
JMJD7	23.81	26	0.55	-0.38	-0.81	0.06
lid	23.85	17	0.82	-0.31	-0.82	0.21
PSR	23.88	26	0.61	-0.28	-0.72	0.15
JMJD4	23.90	31	0.93	-0.24	-0.64	0.17
KDM4A	23.96	63	0.68	-0.18	-0.50	0.13
NO66	24.07	14	0.47	-0.04	-0.60	0.52
w Berlin	24.10	97	0.81	0.00	-0.28	0.28
KDM4B	24.40	25	0.68	0.38	-0.06	0.82
KDM3	25.06	53	2.09	**0.68**	0.34	1.03

genotype	Power (a.u.)	n	sd	Effect Size	Lower CI	Upper CI
KDM2	124	36	44	**0.88**	0.49	1.28
JMJD5	118	58	36	**0.78**	0.44	1.12
JMJD7	112	26	34	**0.60**	0.16	1.04
KDM4A	101	63	32	0.31	-0.01	0.62
KDM4B	99	25	25	0.26	-0.19	0.70
PSR	99	26	36	0.22	-0.21	0.66
JMJD4	94	31	37	0.07	-0.33	0.48
w Berlin	91	97	34	0.00	-0.28	0.28
HSPBAP1	91	43	37	-0.01	-0.37	0.35
NO66	91	14	25	-0.01	-0.57	0.55
KDM3	79	53	31	**-0.38**	-0.72	-0.05
lid	69	17	36	**-0.65**	-1.17	-0.12

genotype	Rhyth-micity (%)	n	Effect Size
KDM4A	100	63	0.5
JMJD4	100	31	0
JMJD7	96	27	0
JMJD5	94	62	0
HSPBAP1	93	46	0
w Berlin	92	105	0
KDM2	92	39	0
PSR	90	29	0
KDM4B	89	28	0
NO66	88	16	0
KDM3	74	71	-0.8
lid	57	30	-1
